# Is This Woman Pregnant?

**Published:** 1873-07

**Authors:** Robert Battey

**Affiliations:** Georgia


### IS THIS WOMAN PREGNANT?

BY ROBERT BATTEY, M.D., OF GEORGIA.

It is related of Mr. Lincoln that he was much in the habit of impressing his ideas upon the minds of his auditors by "telling a little tale." Homely as is this expedient, it has its advantages, and especially when the little tale is veritably drawn from one's own observation and experience.

The yearly augmenting class of women who strive to conceal from their medical attendants the existence of pregnancy for sinister purposes,—the *wouldn't-be accouchèes*,—it is not now my purpose to notice; nor, indeed, those cases of early pregnancy in which the diagnosis presents difficulties to the physician, as well as to the patient herself. I desire, rather, to call attention to a class of cases in which the *good faith* of the patient is not to be impeached; in which the pregnancy, or supposed pregnancy, is far advanced; in which there is no polypus, uterine fibroid or hydatid; in which there can be but one excuse for failure of correct diagnosis, and this simply *inadvertence*. These cases are somewhat rare, and their diagnosis is generally so easy as to make them mortifying to the medical man who carelessly mistakes it. It is, therefore, well to have the attention occasionally drawn to the subject.

My friend, and former teacher, Dr. Ellwood Wilson, of Philadelphia, once related to me from his early practice the following, and I hope I do not transcend the limits of propriety in turning his early experience to useful account at this late day:

Dr. W. had been spoken to and his services engaged early in the supposed pregnancy, perhaps as early as the fifth or sixth month; which was, and as I believe still is, the custom in Philadelphia. The lady was in good society. Dr. W. had visited her repeatedly, as the case progressed, looking after her general health, and had observed nothing unusual, nothing calculated to create suspicion. At length, her full time having expired, her monthly nurse being in attendance, and all the usual paraphernalia of such occasions at hand, Dr. W. was called to the parlor. In due time, the crisis seeming to be impending, he was ushered into the lying-in chamber and made the usual examination, when, to the astonishment of all parties, a puff of wind and collapse of the abdomen dissipated all dreams of pregnancy.

Dr. W. was very unjustly held responsible, by the family and friends, for the mortifying *faux pas*, and suffered for a time the consequences.

Again, perhaps fifteen years ago I was asked by a medical brother to see a patient of his who had a very large ovarian cyst, of which he had been watching the development for some time, and which he contemplated tapping to relieve somewhat the distress of his patient. The lady had been two years bed-ridden, and was much worn down by her sufferings. In placing my hand upon the abdomen, at its lower part, I thought I could feel distinctly, through the attenuated walls, the characteristic tissue of a developed, pregnant uterus, in front of and below the large cyst.

It had not before been thought necessary, under the circumstances of the case, to make an examination *per vaginam.* This was now done, and I found the cervix fully deployed, the os dilated to an inch in diameter, and the fœtal head presenting.

My announcement to the patient of her pregnancy was received with a manifestation of surprise which was only equaled by her indignation. She did not hesitate to pronounce me a blockhead, and to give free expression to her disgust at my temerity in advancing so absurd an opinion. I not only reiterated the opinion already expressed, but rashly predicted that she would be confined within a week.

She was a married woman, living with her husband and a large family of children, some of them nearly or quite grown. She assured me that she had been bed-ridden for two years; that during this period there had been no possible opportunity for her to have become impregnated, and, therefore, that my diagnosis was a physical impossibility. She further stated that she had had no symptoms of pregnancy, and had felt no quickening. Scarce five days elapsed, after my visit, when she gave birth to a still-born child of apparent maturity.

And again, five years ago my services were bespoken to wait upon a lady in an approaching confinement—a lady who had been eight or nine years married and without issue. The infant's wardrobe and other preparations had been timely made, and the attendance of an intelligent monthly nurse engaged.

When called to the labor, I was informed that the pains had been going on quite regularly all day and the previous night; I found them recurring at regular intervals, and becoming quite active and promising. I was glad to learn that "the waters had not yet broken." I instituted an examination to decide if I might not lie down for an hour of rest, and was astonished to find a long and gently tapering cervix uteri, indicative of any thing but parturition. Placing my other hand upon the abdomen, I found the latter but little enlarged, soft and compressible every where, with no sign of either gravid uterus or tumor of any kind. Pressing in the walls of the abdomen strongly, I was enabled to find the fundus of the womb, the cervix being poised upon my right index, and the whole length of the organ was *less than three inches.*

Having satisfied myself fully, I broke the news as gently and considerately as I knew how to the would-be mother, whose chagrin and keenly-felt disappointment I shall not attempt to depict. When the nurse had a little recovered from her dumb astonishment, she addressed me:

"Why, Doctor, you are joking, are you not?"

"No, indeed, I am not."

"Surely, Doctor, you can't be in earnest?"

"Yes, madam, I am in earnest. It is not my habit to deliver my opinions in joke."

"Why, Doctor, you astonish me! Just put your hand upon the abdomen; there, now; don't you *feel* the child move distinctly?"

"Yes, madam, I feel the movements *very distinctly;* and yet there is no child there."

"Now, Doctor, let me lay the abdomen bare, and you shall see for yourself that you are mistaken. There, now; don't you *see* the child kick?"

"Yes, madam, I see the child kick, and yet I *know* that there is no child there."

The deeply mortified wife begged that I would convey the intelligence discreetly to her husband, who was anxiously awaiting, in the parlor, the *denouement.* The pains had now been pretty effectually supplanted, and, administering an anodyne, I left my patient for the night.

There was yet a lingering hope, encouraged by the nurse, that I might, perchance, be mistaken. The pains recurred at intervals, chiefly at night, but gradually wore away as her maternal hopes took their final departure. She has since enjoyed excellent health, and her infantile wardrobe has not yet come into requisition.

Ergo, let us be careful in these matters—careful for our own good name, and careful for the honor and good report of our profession. Let us beware that we do not presume too much upon the knowledge of our patients in such things, even though they be much experienced. Let us decide always for ourselves—let us decide calmly, discreetly and wisely; and let us decide *positively*, ere we venture to speak our mind.

